# Development and Experimental Validation of an Autonomous IoT-Based Monitoring System for Real-Time Water Quality Assessment in the Amazon River

**DOI:** 10.3390/s26123967

**Published:** 2026-06-22

**Authors:** Thiago A. Teixeira, Lennon B. F. Nascimento, Wallace Cavalcante, Ingrid M. F. Ono, Raimundo C. S. Gomes, André L. Printes, Angilberto M. F. Sobrinho, Israel G. Torné

**Affiliations:** 1 Postgraduate Program in Electrical Engineering, State University of Amazonas UEA, Manaus 69050-020, Brazil; wgdsc.mee26@uea.edu.br (W.C.); rsgomes@uea.edu.br (R.C.S.G.); aprintes@uea.edu.br (A.L.P.); asobrinho@uea.edu.br (A.M.F.S.); itorne@uea.edu.br (I.G.T.); 2 Embedded System Laboratory, State University of Amazonas UEA, Manaus 69050-020, Brazil; lbnascimento@uea.edu.br (L.B.F.N.); imfo.pds24@uea.edu.br (I.M.F.O.)

**Keywords:** water quality, autonomous system, Internet of Things, remote monitoring, Amazon River

## Abstract

Monitoring water quality in the Amazon River remains a significant challenge due to limited accessibility, high sediment loads, intermittent connectivity, and the lack of continuous data in remote regions. This study presents the development and experimental validation of an IoT-based system for real-time water quality monitoring. The platform integrates an STM32WL-based embedded architecture with multiparameter sensing, LoRaWAN communication, and configurable monitoring strategies to enable autonomous operation in dynamic environments. The system was validated through a comparative study involving 698 manually collected samples over eight months and 49,570 automated measurements collected during a three-month field deployment. The evaluation considered measurement consistency, variability and operational autonomy based on CONAMA Resolution No. 357/2005. The results showed good agreement between manual and automated measurements, with MAE/RMSE values of 0.18/0.20 °C for water temperature, 0.36/0.44 for pH, and 12.99/20.09 NTU for turbidity. Additionally, the energy analysis demonstrated autonomous operation under variable solar irradiance, achieving self-sufficiency under typical conditions and maintaining operation for up to 4.9 days without solar input. Taken together, the study provides a robust and scalable framework for continuous monitoring in sediment-rich tropical river systems.

## 1. Introduction

Water is one of the most essential natural resources for sustaining life on Earth and plays a central role in human well-being, environmental equilibrium, and economic development. Changes in water quality can directly affect public health and ecosystem stability, making continuous monitoring of water resources a critical requirement for environmental management and sustainable development [1]. In recent decades, increasing environmental degradation and the impacts of climate change have intensified pressures on freshwater systems, reinforcing the need for efficient strategies to support the sustainable use and conservation of water resources [2,3,4].

River basins constitute fundamental spatial units for water resource planning and management because they integrate multiple physical, biological, and anthropogenic processes that directly influence water quality dynamics [5,6]. Water resources are intrinsically connected to environmental components such as soil, vegetation, topography, and land use practices, requiring integrated and interdisciplinary approaches for sustainable management [7,8]. In this context, water quality indicators and environmental assessment methodologies have become strategic tools for evaluating ecosystem conditions, supporting public policies, and guiding evidence-based decision-making processes [9,10,11,12]. These indicators allow large volumes of physicochemical and biological measurements to be interpreted and synthesized into meaningful classifications.

Building on this perspective, recent technological advances have significantly expanded the capabilities of environmental monitoring systems. In particular, the emergence of Internet of Things (IoT) technologies has enabled the development of distributed sensing platforms capable of collecting and transmitting environmental data in near real time [13,14,15,16]. Embedded sensors integrated with wireless communication technologies allow measurements to be performed in remote or difficult-to-access environments, enabling the early detection of environmental changes and supporting timely management interventions. Among the available communication technologies, Long Range (LoRa) has emerged as a promising solution owing to its low power consumption and long transmission range, making it suitable for monitoring applications in remote areas with limited infrastructure [17]. Therefore, the adoption of IoT and LoRa-based monitoring systems represents a viable approach for improving environmental observation capabilities in regions where traditional monitoring infrastructure is limited [18,19].

Despite its global ecological importance, the Amazon Basin faces significant challenges for long-term water quality monitoring. Traditionally, water quality assessment relies on field teams performing in situ measurements and collecting samples for laboratory analyses [20,21]. Although these approaches provide reliable measurements, they are logistically complex, costly, and often result in sparse datasets with limited temporal resolution. In the Amazonian context, the vast spatial scale of the basin, the limited availability of communication and transportation infrastructure, and the difficulty of accessing remote monitoring locations further restrict the implementation of conventional monitoring programs [22].

These limitations highlight the need for monitoring solutions capable of operating autonomously in remote environments while ensuring continuous data acquisition and reliable communication with the monitoring centers [23,24]. Advances in embedded sensing devices, wireless communication systems, and cloud-based data analysis platforms offer promising opportunities to overcome these challenges and expand environmental monitoring capabilities [25]. However, many existing monitoring systems still focus primarily on hardware implementation or short-term experimental deployments, with limited emphasis on methodological frameworks capable of addressing the combined challenges of long-term operation, energy constraints, communication limitations, and environmental variability in highly dynamic and remote tropical river systems.

In particular, monitoring tropical rivers characterized by high sediment loads, strong hydrological variability, intermittent connectivity, and restricted access to energy sources requires monitoring strategies capable of balancing measurement reliability, communication efficiency, and long-term system autonomy. Fixed sampling approaches commonly used in environmental monitoring systems may lead to unnecessary energy consumption during stable conditions or insufficient temporal resolution during periods of rapid environmental change.

To address these limitations, this study presents and experimentally validates a scalable autonomous water quality monitoring framework optimized for high-sediment tropical river systems. The proposed framework integrates configurable monitoring modes, energy-aware embedded architectures, resilient data buffering mechanisms, and long-range LoRa communication to enable continuous monitoring in remote environments with limited infrastructure availability.

The monitoring system was deployed in a real riverine environment in the Amazon region and evaluated through long-term field experiments. This experimental campaign enabled the assessment of measurement reliability, communication stability, and operational performance under real hydrological conditions. By combining configurable operating modes with low-power communication technologies, the proposed framework aims to improve the efficiency and robustness of environmental monitoring in complex tropical river systems.

The main contributions of this study are summarized as follows:

- Development of an embedded sensing architecture enabling continuous monitoring of water quality in remote regions;
- Long-term field deployment of the system in a real-world Amazon River environment, providing experimental evidence of its reliability and operational feasibility;
- Development of a robust mechanical deployment structure capable of supporting long-term operation under sediment-rich Amazon River conditions;
- Energy-aware system design and autonomy analysis, demonstrating sustained operation under variable solar irradiance conditions in remote tropical environments.
- Experimental validation of continuous water quality monitoring under real Amazon River conditions, supporting environmental assessment and water resource management.

The contribution of this study is not centered on a single isolated technological component, but rather on the integration of embedded sensing, long-range communication, robust mechanical protection, data management infrastructure, and continuous field validation in a highly dynamic and sediment-rich tropical environment. This study represents a rare and well-documented case study of an IoT-based environmental monitoring platform successfully deployed under challenging real-world Amazon River conditions.

## 2. Related Work

Water quality monitoring has evolved significantly through the integration of sensor networks, Internet of Things (IoT) architectures, and data-driven analytical techniques. However, despite these technological advances, important methodological and validation gaps still remain in the literature.

Early studies primarily focused on understanding the geographic distribution of monitoring efforts and the influence of environmental factors on water quality. Misuikas et al. [26] highlighted the concentration of research in already monitored regions, leaving remote and hydrologically complex areas underrepresented. Similarly, Cappello et al. [27] demonstrated community-driven forest monitoring approaches in Peru, reinforcing the need for scalable solutions in low-infrastructure environments.

Several studies have explored physicochemical parameter monitoring and sensor integration [28,29]. These studies emphasize parameters such as pH, turbidity, conductivity, dissolved oxygen, and temperature, and discuss the integration of AI and ICT technologies for improved analysis. Nevertheless, most investigations either provide high-level reviews or focus on parameter selection without proposing validated autonomous field-deployable frameworks for their implementation.

Conventional water quality studies [22,30,31,32,33,34] frequently rely on manual sampling, laboratory-based validation, and secondary public datasets. Although these approaches contribute to environmental understanding, they lack continuous real-time acquisition and autonomous in-situ sensing capabilities, particularly in remote riverine environments. In addition, validation is often limited to statistical analysis of historical datasets rather than experimental comparisons against commercial-grade instrumentation.

Recent IoT-based monitoring systems have introduced wireless communication and real-time transmission capabilities [35,36,37,38,39,40,41,42]. Surendran et al. [35] proposed an IoT platform with industrial sensors and GSM communication; however, the hardware implementation details and statistical validation were limited. Alghamdi et al. [36] evaluated the LoRaWAN communication performance exclusively through simulations without real environmental deployment. Moparthi et al. [38] implemented M2M communication but did not include configurable acquisition intervals. Yu et al. [40] described the system architecture, but the validation remained laboratory-constrained. Luo et al. [42] focused on predictive models derived from secondary data without developing proprietary sensing hardware.

More recent approaches have incorporated advanced data processing and mobile sensing strategies. For example, Islam et al. [43] proposed an IoT-based system integrated with machine learning models for water quality prediction, achieving high classification accuracy. However, the system relies on Wi-Fi communication and focuses on predictive tasks rather than continuous long-term monitoring under constrained connectivity conditions.

Similarly, Lopez et al. [44] developed a dynamic wireless sensor network combining mobile and fixed nodes for spatial monitoring and real-time visualization. Despite its flexibility, the approach emphasizes network architecture without addressing long-term autonomous operation or validation against reference instruments. Additionally, it does not include key parameters such as electrical conductivity and turbidity.

Although these works demonstrate important progress, three major gaps can be identified:

1. Limited long-term field validation under hydrologically complex environments, particularly in high-sediment tropical rivers.
2. Limited support for remotely configurable acquisition and communication parameters during long-term field deployments.
3. Insufficient discussion of buffering and fault-tolerant data retention strategies in scenarios with unstable connectivity.

These limitations highlight the need for experimentally validated monitoring frameworks capable of long-term autonomous operation, reliable communication, and sustained deployment in remote riverine ecosystems. Table 1 summarizes the main characteristics of the related works and highlights the research gaps addressed by the proposed monitoring framework.

**Table 1:** Methodological comparison of related works (focus on autonomous monitoring frameworks).

Work	Field Deploy	Long Term	Ref. Stats	Config. Sampling	Fault Aware
[34]	Yes	Partial	No	No	No/ND
[35]	Yes	No	No	No	No/ND
[36]	No	No	No	No	No (ND)
[37]	Yes	No	No	No	No/ND
[38]	Yes	No	No	No	No/ND
[39]	Yes	No	No	No	No/ND
[40]	No	No	No	No	No (lab)
[41]	Yes	No	No	No	No/ND
[42]	No	No	No	No	No (sec)
[43]	Yes	No	No	No	No/ND
[44]	Yes	No	No	No	No/ND
This work	Yes	Yes	Yes	Yes	Yes

Field Deploy: real-world deployment; Long Term: multi-week/month operation; Ref. Stats: quantitative validation vs commercial/reference probe (e.g., RMSE/MAE/Bias/correlation); Config. Sampling: Configurable platform; Fault Aware: buffering packets/fault-tolerance. ND: not detailed; sim: simulation-only; lab: laboratory-only; sec: secondary/public data.

## 3. Materials and Methods

### 3.1. Monitoring Device Architecture

This section outlines the methods employed to develop a remote water quality monitoring system for river environments. This study implements a Water Quality Monitoring System (WQMS), a technological platform designed to collect, record, and analyze the physical and chemical properties of water in real time and at predefined periodic intervals, within the context of the Internet of Things (IoT). The proposed monitoring platform presents an alternative method for collecting water quality indicators from rivers. Accordingly, the system was designed to sample water temperature, electrical conductivity, dissolved oxygen, pH, and turbidity data. These parameters were chosen because of their direct relevance in assessing the health of aquatic ecosystems and detecting contamination. These indicators are widely used to identify physical and chemical changes in aquatic environments, enabling a comprehensive assessment of water conditions and supporting informed decision-making in water resource management.

**Figure 1:**
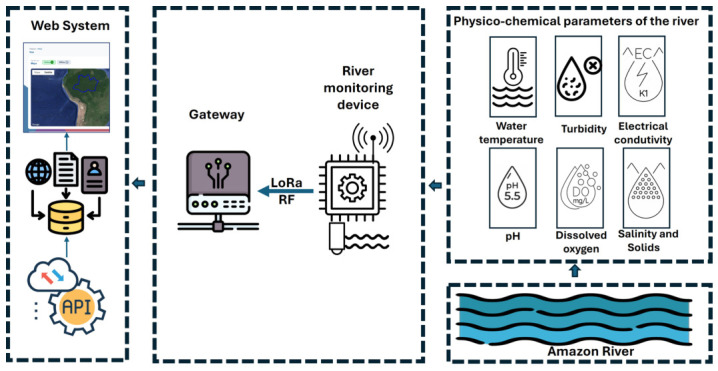
Representation of the architecture of the proposed monitoring system, showing the sensing device deployed in the river, from the sensor array to the communication interface.

The architecture of the proposed system is organized into distinct functional segments: the mechanical enclosure, responsible for protecting and positioning the sensors; the sensing module, which collects the physical–chemical parameters of the water; the hardware layer, which encompasses all the peripherals used in the solution; the firmware layer, which defines the implemented control logic; and the data processing and communication layer, responsible for transmitting the acquired data to the network server. Figure 1 presents an integrated overview of the system architecture and its operations. This architecture allows continuous monitoring, even in remote environments where traditional communication infrastructure is unavailable.

### 3.2. Sensing System Hardware

**Figure 2:**
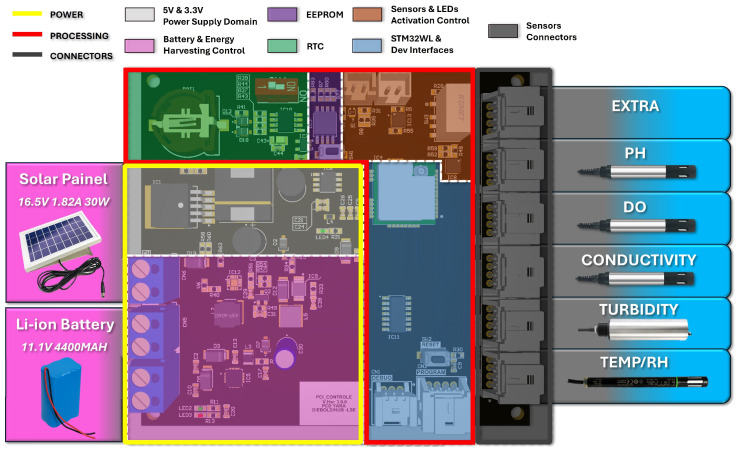
Hardware architecture of the monitoring system, highlighting the functional blocks of the PCB, including power supply and energy harvesting, battery management communication interfaces, and sensor connectors.

The proposed hardware architecture was designed for low-power IoT applications and to support autonomous operation in remote locations. Therefore, the circuit was divided into two fundamental cores: a power supply module and a processing control module that integrates a printed circuit board (PCB). Figure 2 shows the sectors of the proposed hardware for the monitoring system.

The processing and control module features a microcontroller from the STM32WL family, developed by STMicroelectronics [45]. As shown in Figure 2, the processing module concentrates most of the utility resources of the system hardware, divided into programming and debugging interface sectors, communication buses, and auxiliary circuits.

Among these sectors, the I2C (Inter-Integrated Circuit) communication bus stands out, interconnected with the integrated circuits (ICs) of non-volatile memory Electrically Erasable Programmable Read-Only Memory (EEPROM), which support the processes of transmitting and storing measurement messages, and Real-Time Clock (RTC) providing time reference for the system. In addition, a Watchdog Timer (WDT) was included to protect against potential system crashes.

The power supply module was designed to provide hybrid power, allowing the system to be powered by an electrical grid near the monitoring site, which offered stability to the system. The other power mode consists of an energy harvesting circuit based on photovoltaic energy that can meet the system’s power requirements in remote or hard-to-reach locations.

Both power supply modes were designed to provide 12 V DC as the input voltage for the system. In this way, the system can be powered through the utility grid, where the voltage conversion is carried out by a 127 V AC to 12 V DC *colmeia* power supply. Nevertheless, it can also be powered through a photovoltaic energy harvesting circuit, which supports the circuit and battery charging, and routes the voltage level to a step-up circuit to 12 V DC. This voltage is used to power the sensors and is supplied to a voltage regulator circuit that provides 5 V for the operation of the board resources and 3.3 V for powering the control module.

#### 3.2.1. Sensing Layer and Sensor Selection

The sensor layer of the data collection system comprises water quality measurement probes and a climate sensor connected to the printed circuit board (PCB). The proposed system architecture was designed to allow the measurement of the following water quality parameters: pH, dissolved oxygen, electrical conductivity, turbidity, and water and air temperature.

One of the main purposes of this monitoring device is to provide an alternative method for collecting data on the water quality of river basins. Therefore, it is necessary to employ measurement devices with accuracy comparable to that of the tools traditionally used in field-based water quality analysis. Therefore, a set of probes from the manufacturer BOQU Instruments [46] was used, the company is specialized in the production of probes, meters, and water quality analyzers at the laboratory level. Accordingly, probes with encapsulation suitable for full immersion in a liquid environment were selected for the study. The accuracy specifications of the probes are listed in Table 2.

Each water quality probe included in the sensing layer was carefully selected according to the monitored parameter to ensure accurate and reliable measurements. Different sensing principles were employed depending on the analyzed variable. For parameters such as turbidity and suspended particles, optical sensors were used to detect light dispersion and absorption in the water sample. For chemical parameters, including pH and dissolved oxygen, electrochemical probes with selective membranes enabled controlled reactions with the target substances, generating signals proportional to their concentration. For electrical measurements, such as conductivity, dedicated analog-to-digital conversion (ADC) circuits were employed to convert the analog signals from the electrodes into digital values processable by the monitoring platform.

This combination of sensing technologies ensured that each water quality parameter was measured using the most suitable acquisition method, improving both measurement accuracy and operational reliability. In addition, all selected probes communicate through the RS-485 standard using the Modbus RTU protocol, enabling simultaneous communication with multiple devices and ensuring stable and consistent data transmission.

**Figure 3:**
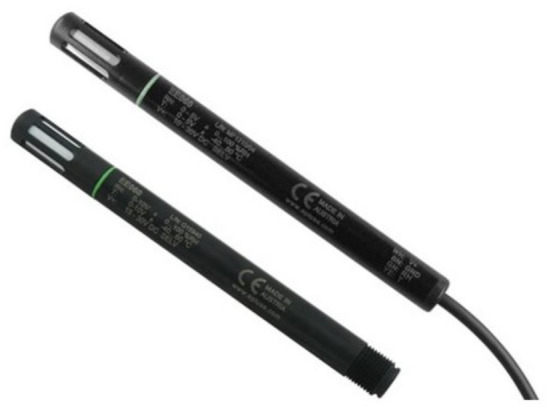
EE060 Temperature and Relative Humidity Sensor.

To complement the sensing layer of the monitoring system, an EE060 climate sensor was integrated into the platform, as illustrated in Figure 3. This sensor measures air temperature and relative humidity at the monitoring site, providing contextual environmental information that supports the interpretation of the collected water quality data.

#### 3.2.2. Sensor Calibration and Validation Procedures

Before installing the monitoring device in the Amazon River, all the sensors integrated into the platform were calibrated to ensure the accuracy and reliability of the measurements. Calibration is a critical step in environmental monitoring systems, because each sensor has specific response and sensitivity characteristics that can introduce deviations in measurements if not properly adjusted. Furthermore, environmental conditions can amplify small inaccuracies in measurements, making proper calibration essential for obtaining reliable field data.

**Figure 4:**
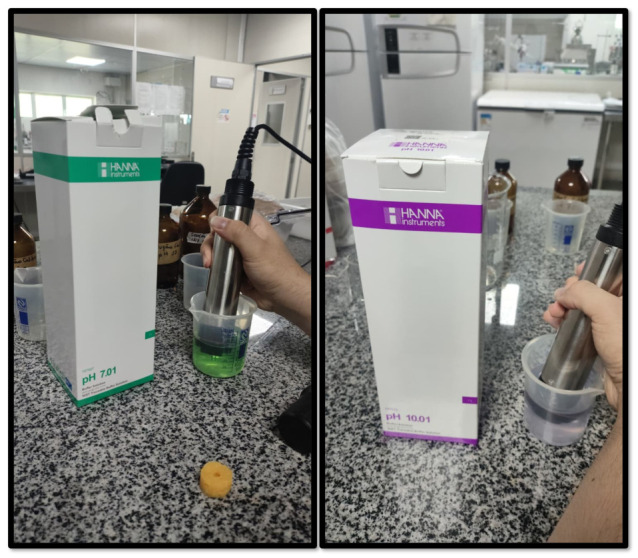
pH sensor calibration process.

All calibration procedures were performed under controlled laboratory conditions, using certified reference standards for each monitored parameter. The procedures adopted are described below:

- pH: Calibrated using buffer solutions with known pH values of 4, 7, and 10, covering the expected operating range of the sensor. Figure 4 shows the reference solutions used during the calibration procedure.
- Turbidity: Calibrated using suspended particle standards that simulate different turbidity levels, allowing adjustment of the optical sensor response across the entire measurement range.
- Electrical Conductivity: Calibrated and verified using saline solutions with known conductivity values (1413 µS/cm), ensuring accurate measurements under different salinity and total dissolved solid conditions.
- Dissolved Oxygen: Calibrated using saturated and deoxygenated solutions to ensure accurate measurements in critical dissolved oxygen concentration ranges.

In addition to the initial calibration performed before deployment, periodic recalibration procedures were scheduled every six months to compensate for potential sensor drift and ensure long-term measurement stability during field operation. These calibration procedures ensured that all sensors operated within their specified measurement ranges before field deployment, improving the reliability of environmental data collected during the monitoring campaign.

After calibration, the sensors were verified for repeatability and stability of the readings, ensuring that small fluctuations did not affect continuous data collection in the river. The calibration process also included the validation of communication and integration with the control PCB, guaranteeing that all sensors transmitted data correctly to the system before installation.

### 3.3. Mechanical Design for Deployment in the Amazon River

The Amazon River presents challenging environmental conditions for long-term deployment of autonomous monitoring systems. Strong water currents, reaching velocities of up to 7 km/h, combined with high sediment concentrations, floating debris, dense riparian vegetation, seasonal water level variations, and temperature fluctuations, can compromise the structural stability and operational reliability of sensing devices deployed in riverine environments.

**Figure 5:**
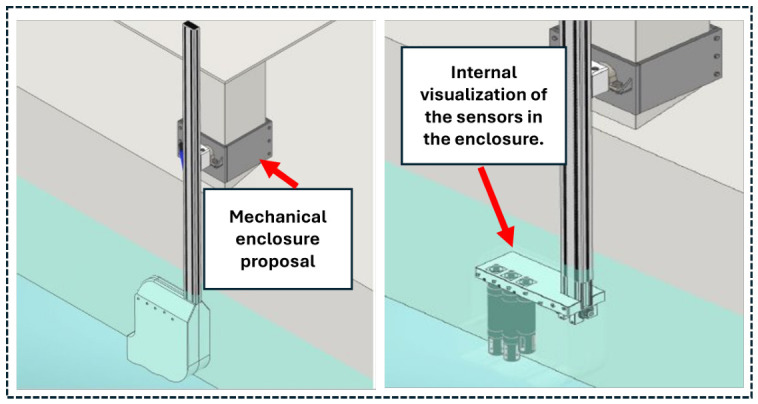
Proposed mechanical enclosure designed to protect and integrate the water quality monitoring sensors under field conditions.

To address these challenges, a robust mechanical enclosure was developed to protect the sensing components while maintaining direct contact between the sensors and the surrounding water body. As illustrated in Figure 5, the proposed structure was designed to withstand mechanical stress, sediment accumulation, debris impact, and biological interference typically observed in large tropical river systems. The enclosure also supports stable sensor positioning during continuous operation under variable hydrodynamic conditions.

The mechanical structure was additionally designed to facilitate deployment in different monitoring scenarios along the Amazon River, including ports, riverbanks, and floating support structures. This flexibility is particularly important in remote regions with limited infrastructure availability, where monitoring stations must be installed under diverse environmental and operational conditions.

Overall, the proposed mechanical design provides structural stability, environmental protection, and operational reliability, supporting the continuous acquisition of water quality data during long-term field deployment in the Amazon River.

### 3.4. Firmware Operational Architecture

The firmware architecture of the monitoring device was developed for low-power operation and implemented on the STM32WL microcontroller platform. The system relies on the utility software components provided by the manufacturer, which include support for the LoRaWAN communication stack, low-power operating modes, and an event-driven task management framework. This software infrastructure enables the efficient coordination of sensing, processing, and communication tasks while minimizing energy consumption.

Within this architecture, the system operation is organized using a set of routines responsible for sensing, system supervision, and communication management. The main routines implemented in the firmware are as follows:

- Measurement Routine: Responsible for the periodic acquisition of water quality parameters from the connected sensors. During execution, the sensors are activated and sampled, and the collected measurements are stored in the system memory as a structured data packet. Subsequently, transmission attempts are performed through the LoRaWAN interface to send the generated packet to the network server. If the transmission is unsuccessful, the packet is temporarily stored in local memory for future communication attempts.
- Keep-Alive Routine: Responsible for maintaining periodic communication with the network server. When executed, the routine first checks for previously stored messages that were not transmitted successfully and prioritizes their transmission. If no pending messages are found, a simplified keep-alive message is sent to indicate that the device is operational.
- Watchdog Routine: Maintains system reliability by periodically feeding the watchdog timer, preventing software lockups and enabling automatic system recovery in the event of failures.

**Figure 6:**
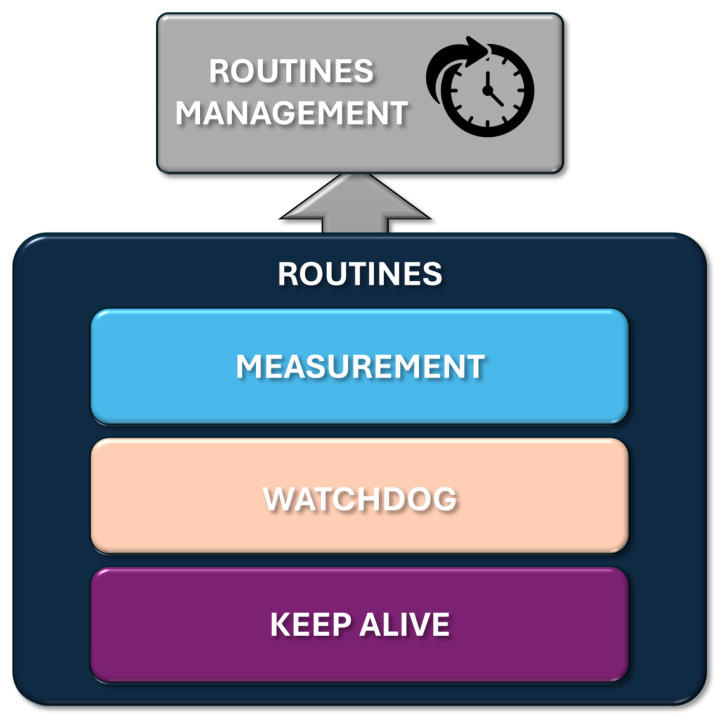
Event-driven firmware routines implemented in the monitoring device.

Figure 6 summarizes the set of routines implemented in the firmware and illustrates the functional tasks responsible for the continuous operation of the system, which are managed by an event-driven manager framework.

The firmware was developed using STM32CubeIDE version 1.16.1 (STMicroelectronics). The STM32WL software framework adopts an event-driven execution model in which each task is associated with a priority level and triggered by specific system events. These events are typically generated by periodic timers and posted to the sequencer module. The sequencer is responsible for managing the execution of registered tasks and, activating them according to the events received. This mechanism allows the coordination of multiple routines while maintaining an efficient task scheduling.

**Figure 7:**
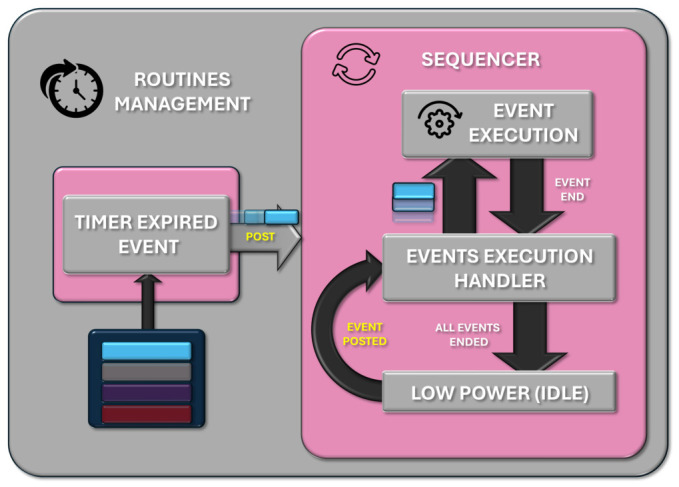
Sequencing Structure.

After a task is completed, the sequencer verifies whether any additional events are pending. If no events remain, the microcontroller transitions to a low-power mode and remains idle until a new event is generated. Figure 7 illustrates this execution flow, showing how timer-triggered events are posted to the sequencer, followed by task execution and transitions to low-power operation.

#### Data Management and Communication

A key concern during device development was the potential loss of measured data when the control PCB was unable to establish communication with the network server. To prevent this, a dedicated storage mechanism for the measured data was implemented.

**Figure 8:**
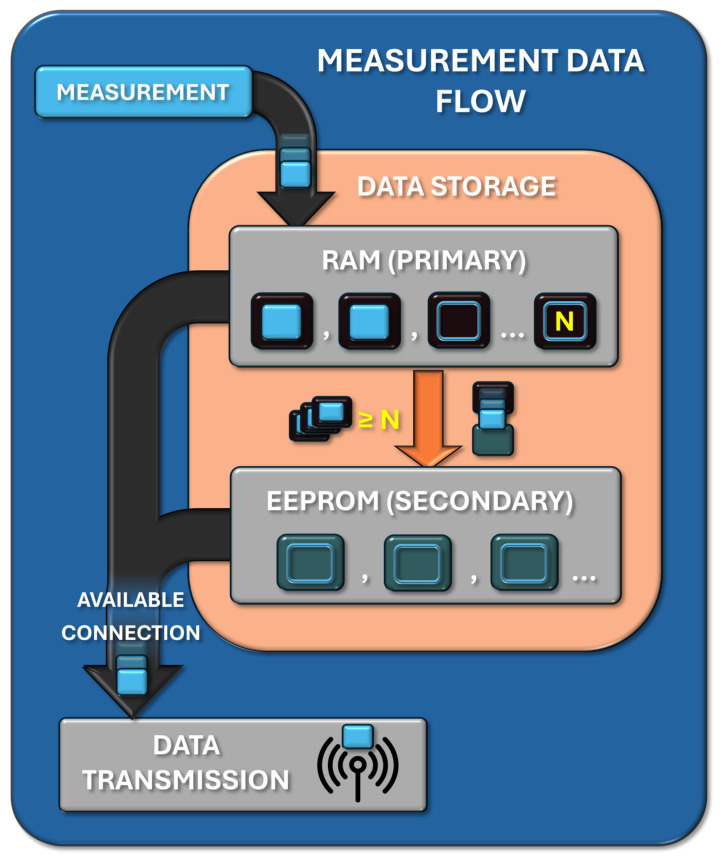
Message transmission cycle for the device module.

As shown in Figure 8, the storage system operates in two stages. The measurement routine stores the collected sensor data as structured packets in the RAM and immediately attempts to transmit them through the LoRaWAN interface. If the transmission fails, the packets are temporarily stored in the RAM during subsequent communication attempts.

When the RAM buffer reaches its capacity owing to the lack of connection with the network server, the pending packets are transferred to the EEPROM memory, allowing the measurement data to be preserved for later transmission. This buffering procedure, first in the RAM and subsequently in the EEPROM, reduces the number of write cycles in the EEPROM and extends its operational lifetime.

The keep-alive routine, periodically checks whether the stored packets are available in the system memory. If pending messages are detected, the routine prioritizes their transmission when a communication link becomes available.

### 3.5. LoRaWAN Communication

To enable long-range communication with low energy consumption, the monitoring device employs the LoRaWAN protocol. LoRaWAN is a Low Power Wide Area Network (LPWAN) technology designed for long-distance communication between distributed sensor nodes and a central network infrastructure. In this architecture, the monitoring device operates as an end-node that periodically transmits measurement data through LoRa radio packets. These packets were received by a LoRaWAN gateway, which forwarded the data to the network server and subsequently to the application server responsible for data visualization and analysis.

Uplink transmissions occur during the execution of measurement and keep-alive routines. During these communication windows, the system can receive downlink messages from the network. Once a transmitted packet is acknowledged by the gateway, the corresponding message is removed from local storage.

Downlink messages are also used to deliver configurable parameters to the device, such as sampling intervals and threshold ranges, allowing the monitoring behavior to be adjusted remotely.

LoRaWAN was selected due to its ability to provide long communication range with low power consumption, which makes it suitable for environmental monitoring applications where devices are deployed in remote locations and must operate for extended periods without maintenance.

### 3.6. Application Server and Web-Based Monitoring Platform

During the transmission process, the collected data are sent from the monitoring device to the nearest LoRaWAN gateway. Upon reception, the gateway forwards the packets to the network server using the User Datagram Protocol (UDP), enabling communication with the application layer. In this study, the application server is based on ChirpStack LoRaWAN [47], an open-source platform widely adopted in LPWAN deployments. ChirpStack provides essential functionalities such as device management, secure authentication, payload decoding, data storage, and integration with external applications through standardized APIs.

Building on this infrastructure, a web-based application layer was developed to enable real-time visualization and analysis of the collected environmental data. Through API integration, the decoded data are made available to a user interface that presents both real-time measurements and historical trends. The application supports continuous monitoring of water quality parameters, facilitating the identification of temporal patterns and potential anomalous events. This architecture enhances data accessibility and supports remote monitoring while maintaining scalability and flexibility for future expansion of the monitoring network.

### 3.7. Configurable Monitoring Strategy

Additionally, environmental monitoring systems deployed in riverine environments must operate under varying conditions related to energy availability, communication constraints, and environmental dynamics. To address these requirements, the proposed platform implements a configurable monitoring strategy based on remotely adjustable parameters and threshold-based event handling mechanisms.

Through the web application, users can remotely configure key system parameters, including sensor sampling frequency, communication intervals, and acceptable threshold ranges for each monitored water quality variable. These parameters are transmitted to the device and stored locally, allowing the operating behavior of the monitoring system to be adjusted according to the characteristics and requirements of each deployment scenario.

Based on these configurations, the monitoring device operates according to three distinct modes designed to balance temporal resolution and energy consumption:

- Operating Mode 1: Designed for installations connected to the local power grid. In this mode, shorter acquisition and communication intervals are adopted, enabling more frequent sensor readings and LoRaWAN transmissions. A minimum transmission interval of two minutes is maintained to avoid excessive use of the shared communication channel.
- Operating Mode 2: Intended for remote deployments powered exclusively by batteries. In this configuration, longer acquisition and transmission intervals are used to reduce energy consumption and extend device autonomy.
- Threshold Alert Mode: Activated whenever a monitored parameter exceeds predefined threshold limits. In this condition, the system immediately transmits an alert message to the server and temporarily increases the acquisition and transmission frequency, allowing higher temporal resolution during significant environmental events.

Once the monitored parameters return to their expected ranges, the system restores the default operating configuration defined for the deployment. This configurable monitoring strategy improves energy efficiency under stable environmental conditions while maintaining the capability to generate alerts and increase temporal resolution during relevant environmental variations.

### 3.8. Experimental Deployment

To evaluate the operational performance of the proposed monitoring solution under real environmental conditions, the system was deployed in the Amazon River region, specifically in the city of Parintins, located in the state of Amazonas, Brazil. The monitoring device was installed on a barge at the Port of Parintins, allowing continuous access to river water and providing a suitable platform for long-term environmental monitoring.

**Figure 9:**
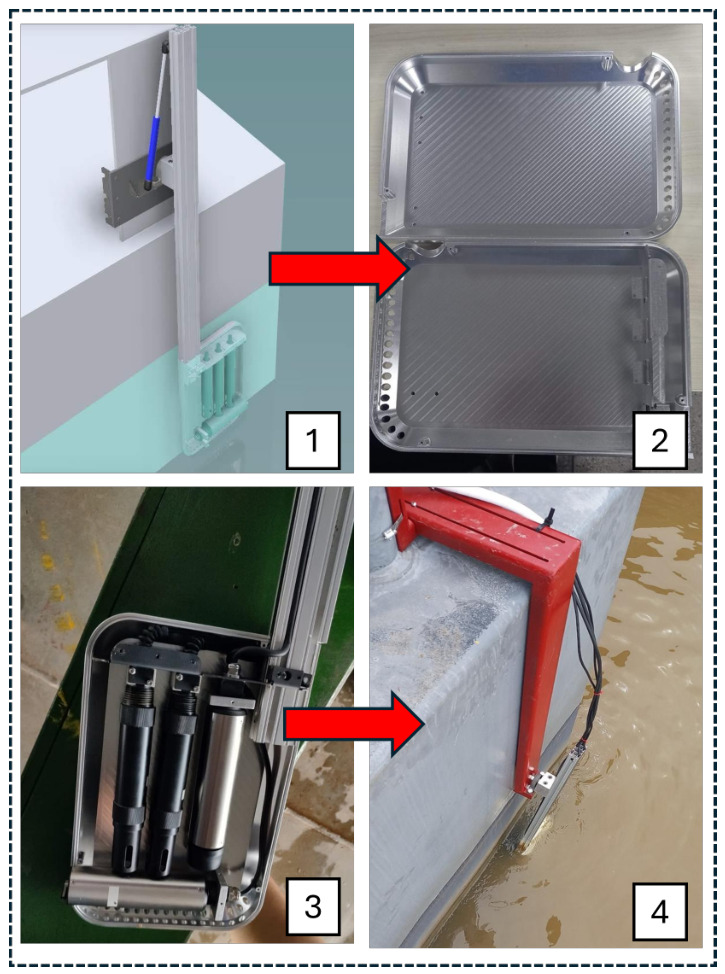
Stages of the mechanical enclosure design and implementation. (1) Conceptual mounting structure; (2) completed enclosure; (3) internal assembly with integrated sensors for data acquisition; (4) field installation under real environmental conditions.

During the deployment phase, the mechanical structure designed to protect the sensing components was positioned in the river to ensure proper immersion of the sensors at a depth of approximately 70 cm, allowing the monitoring of surface water quality. The structure also provided protection against environmental stressors such as water currents, floating debris, and sediment accumulation. Figure 9 illustrates the installed mechanical structure and the positioning of the monitoring device in the aquatic environment.

**Figure 10:**
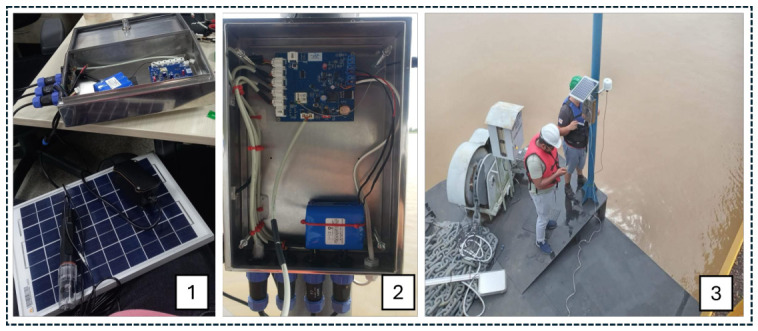
Hardware assembly and field installation of the monitoring system. (1) Initial system configuration; (2) Internal configuration of the box, showing the electronic circuit, battery and wiring; (3) Installation of the system on the barge structure under real environmental conditions.

The hardware installation of the system, illustrated in Figure 10, responsible for communication and data acquisition from the sensors, was carried out on the barge’s lighting pole structure, which served as a stable support point for the device. This configuration allowed for the secure attachment of the hardware system, ensuring structural stability under field conditions. Furthermore, the selected installation point facilitated access for maintenance operations, contributing to the reliability and continuity of the monitoring process.

**Figure 11:**
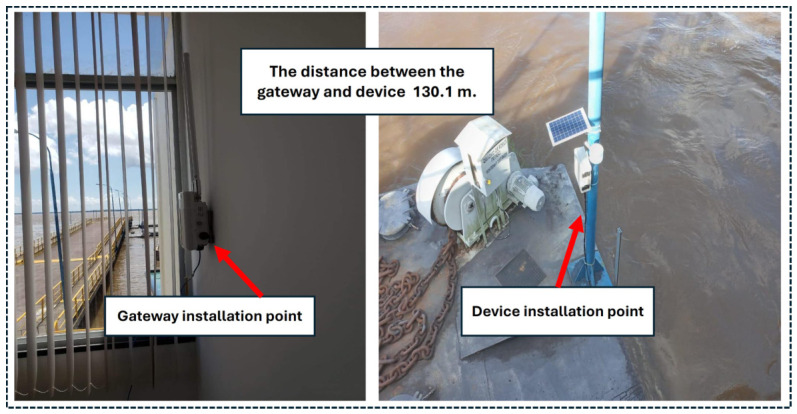
Device and gateway installation site.

**Figure 12:**
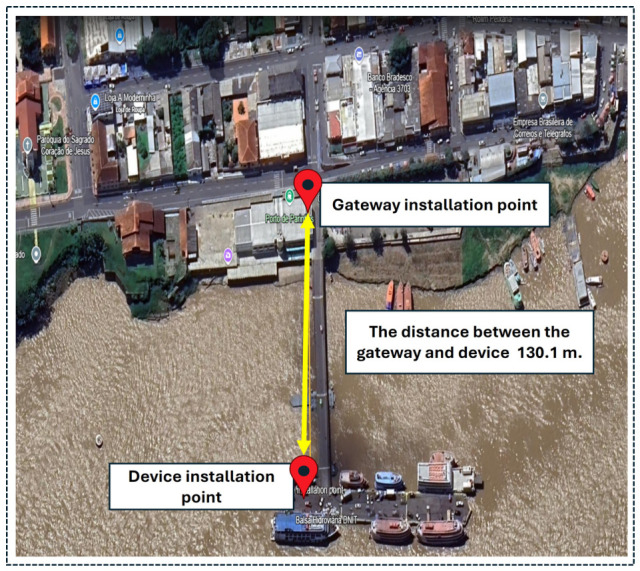
Deployment layout of the monitoring system, illustrating the installation points of the LoRaWAN gateway and the river monitoring device, along with the communication distance between them (130.1 m).

To enable wireless data transmission, a LoRaWAN gateway was installed approximately 130 m from the monitoring prototype. The gateway was responsible for receiving the data transmitted by the device and forwarding it to the network server for storage and analysis. Figure 11 and Figure 12 illustrate the installation environment and the relative positioning of the device and the communication infrastructure.

The experimental site presents several connectivity challenges typical of remote regions in the Amazon. The city of Parintins faces limitations in telecommunications infrastructure, including restricted availability of mobile and high-speed internet services, as well as variable signal quality. These conditions represent realistic operational scenarios for environmental monitoring systems deployed in remote riverine environments.

Despite these connectivity constraints, the developed monitoring prototype successfully performed automated data acquisition and transmission, enabling continuous monitoring of water quality parameters in the Amazon River under real operating conditions.

Additionally, during the deployment phase, an issue was identified with the dissolved oxygen (DO) sensor, which prevented the acquisition of reliable automated measurements for this parameter. As a result, continuous DO data could not be collected by the proposed system throughout the monitoring period.

A preliminary analysis indicates that the most probable failure mode is associated with electronic instability during the communication testing stage performed before field deployment. Specifically, possible grounding (GND) inconsistencies in the communication interface may have affected the electronic circuitry of the sensing module, thereby compromising its operation.

Other potential causes, such as biofouling, membrane degradation, or improper installation conditions, were considered less likely, as the sensor did not exhibit progressive signal drift or gradual degradation patterns typically associated with these mechanisms. Instead, the observed behavior was consistent with an abrupt loss of functionality, supporting the hypothesis of an electrical failure.

### 3.9. Statistical Evaluation Metrics

**Figure 13:**
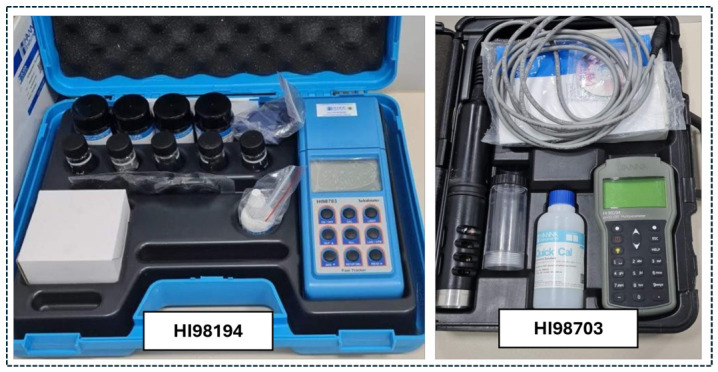
Commercial instruments used as reference devices: HI98194 multiparameter probe and HI98703 turbidity meter.

To evaluate the performance and reliability of the proposed monitoring system, a comparative validation approach was adopted. Commercial instruments were used as reference devices, including the HI98194 multiparameter probe (Hanna Instruments, Woonsocket, RI, USA) for pH, electrical conductivity, and dissolved oxygen measurements, and the HI98703 turbidimeter for turbidity assessment. Both instruments, manufactured by Hanna Instruments, are illustrated in Figure 13.

The reference dataset was obtained through manual measurements using calibrated commercial instruments over a period of approximately eight months, providing a baseline characterization of water quality parameters under real environmental conditions. Subsequently, the proposed monitoring system was deployed at the same location, and automated measurements were collected continuously over a period of approximately three months, enabling high-resolution temporal monitoring and direct comparison between the two datasets.

It is important to note that the difference in the temporal intervals between the manual and automated datasets is related to the data acquisition strategy. The manual sampling protocol was initiated before the deployment of the proposed system, with the objective of building a reference dataset. Due to the development and field deployment timeline, the automated monitoring period does not fully overlap with the entire manual measurement interval, and therefore the time windows are not the same. Additionally, the deployment of the automated system was authorized for a limited period of three months on a floating platform at the Port of Parintins, managed by the National Department of Transport Infrastructure (DNIT). This limited deployment window was possible because the barge used for installation was temporarily out of operation due to maintenance activities. After this period, continued deployment became impractical due to the high traffic of vessels docking at the port, which would compromise the safety of the monitoring system. For this reason, the measurements presented in this study correspond to the year 2022, during which both manual and automated data acquisition were conducted under real field conditions. As a result, the comparative analysis was conducted using only the subset of temporally aligned observations, ensuring a consistent and fair evaluation between the two approaches.

The evaluation methodology focused on three main aspects:

- Consistency analysis: to assess whether the monitoring system was capable of reproducing stable measurement patterns over time under similar environmental conditions.
- Variability analysis: to evaluate the natural fluctuations of the monitored parameters and the ability of the system to continuously capture these variations under real environmental conditions.
- Comparative analysis: to evaluate the agreement between the reference measurements and the data obtained by the automated monitoring system through statistical comparison metrics.

To support the interpretation of the monitored parameters and the identification of anomalous conditions, threshold values were defined based on the Brazilian environmental regulation CONAMA Resolution No. 357/2005, classification II of rivers. This regulation establishes water quality classification criteria and defines acceptable limits for key physicochemical parameters, including pH, dissolved oxygen, turbidity, and electrical conductivity, according to different water use classes. The reference limits adopted in this study are summarized in Table 3.

#### 3.9.1. Mean Absolute Error

The Mean Absolute Error (MAE) is a metric that quantifies the average magnitude of errors in a set of predicted values. The mean absolute error can be calculated using Equation (1).

(1) $$MAE=\frac{1}{n}\sum_{i=1}^{n}\left|y_{i}^{\mathrm{Hanna}}-y_{i}^{\mathrm{WQMS}}\right|$$

#### 3.9.2. Root Mean Square Error

The Root Mean Square Error (RMSE) is a metric derived from the mean square error, representing the square root of the average of the squared differences between the estimated and the actual values. This metric provides an error measure in the same unit as the observed variable, facilitating interpretation and comparison of the estimation performance.

The RMSE can be obtained from Equation (2).

(2) $$RMSE=\sqrt{\frac{1}{n}\sum_{i=1}^{n}{\left(y_{i}^{\mathrm{Hanna}}-y_{i}^{\mathrm{WQMS}}\right)}^{2}}$$

#### 3.9.3. Bias

The Bias is a metric that quantifies the average systematic difference between two sets of measurements. It indicates whether one system tends to consistently overestimate or underestimate the values in relation to a reference.

The bias between the reference measurements (Hanna probe) and the proposed system (WQMS) can be obtained from Equation (3).

(3) $$\mathrm{Bias}=\frac{1}{n}\sum_{i=1}^{n}\left(y_{i}^{\mathrm{WQMS}}-y_{i}^{\mathrm{Hanna}}\right)$$

## 4. Results

### 4.1. Analysis of Normal and Anomalous Conditions

A historical dataset obtained from manual measurements was used to characterize the baseline behavior of water quality parameters in the monitored region. Approximately 698 measurements were collected over an 8-month period. Additionally, the automated monitoring system was deployed for three months, during which a total of 49,570 measurements were continuously acquired. All samples were analyzed based on the reference limits defined by CONAMA Resolution No. 357/2005 (Class II) and classified as normal or anomalous, as summarized in Table 4.

### 4.2. Integrated System Performance

**Figure 14:**
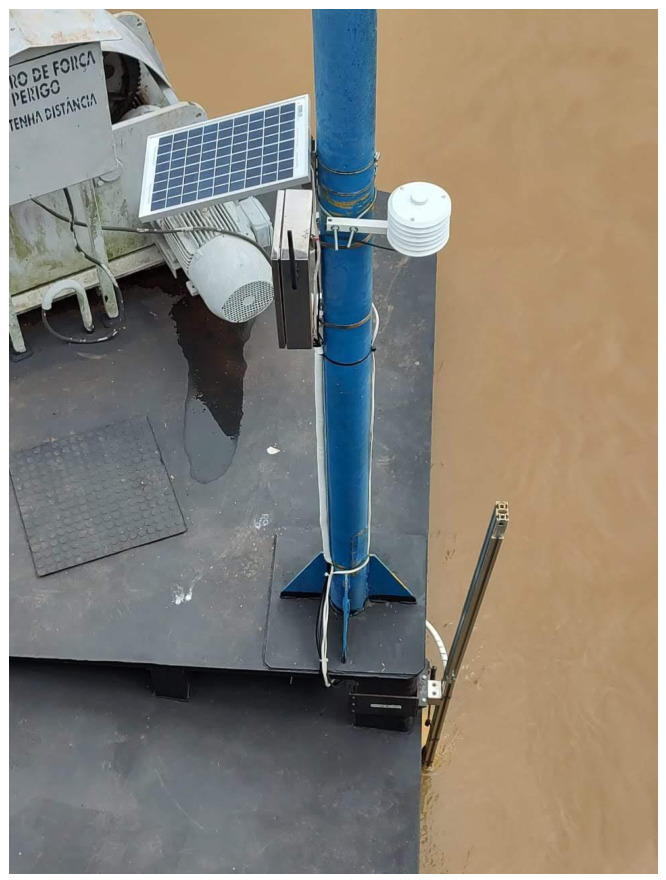
Complete installation of the monitoring system at the Port of Parintins, Amazon River.

The installed system is shown in Figure 14. Throughout the entire operational period, no signs of internal condensation, component oxidation, or failures associated with water ingress were observed in the external enclosure housing the system hardware. These issues are commonly reported in devices deployed in tropical aquatic environments.

In addition, the mechanical structure, illustrated in Figure 9, demonstrated the ability to withstand occasional impacts from branches and small floating debris without structural deformation or functional damage. The internal arrangement of the sensors promoted uniform water circulation, preventing stagnation and ensuring that each measurement accurately represented the environmental conditions.

During the testing period, the platform maintained stable communication with the central system, demonstrating robustness even under signal variability. This ensured continuous data acquisition and transmission without the need for frequent manual interventions.

From a maintenance perspective, the modular design proved to be efficient, allowing rapid access to sensors and acquisition boards. This facilitates targeted inspections and interventions without interrupting the monitoring process.

### 4.3. System Autonomy

The system autonomy is determined by the energy consumption across three states: Measurement, keep alive transmission, and sleep. To estimate the power demand of each state, the current draw was measured at the nominal battery voltage of 11.1 V. Table 5 summarizes the results. Measurement is the most power-intensive state, drawing 185 mA over 60 s to acquire sensor data. Although transmission operates at 100 mA, its shorter duration of 1 s results in a lower overall energy contribution. The sleep state represents the primary driver of daily energy consumption, as the system remains in this condition during all periods without active measurement or transmission. The achieved sleep current of 20 mA corresponds to the consumption of the entire system in sleep mode, including all embedded components and peripheral circuits, and not only the microcontroller. This value directly governs long-term autonomy and is therefore the most critical parameter in the energy budget.

The system can be configured to execute different numbers of measurements and transmissions per activity cycle. Therefore, the overall energy consumption depends directly on the frequency of these operations throughout the day. The system autonomy was estimated with the real measured currents presented in the Table 5, using the per-process energy contribution to derive the total hourly power demand. The analysis was conducted by defining the number of measurements and Keep Alive transmissions per hour, computing the associated energy for each state, and aggregating the results to obtain the daily energy consumption.

The results are presented in Table 6, where energy consumption is expressed in watt-hours per hour (Wh/h) and per day (Wh/day), for each operating mode. In OP1 mode, which assumes connection to the local power grid, a sampling rate of 30 measurements per hour (one every two minutes) was adopted, as the unrestricted power availability allows for higher acquisition frequency. In OP2 mode, designed for battery-powered operation, the sampling rate was reduced to 6 measurements per hour (one every ten minutes) to preserve energy. In both Alert variants, the sampling rate is doubled upon detection of measurements exceeding predefined threshold limits. Given its comparatively low energy impact, the Keep Alive transmission rate was fixed at 30 transmissions per hour across all operating modes.

Table 6 presents the daily consumption of the system under battery-powered operation with reduced process frequency. Among the battery-powered modes, OP2 presents the lowest daily consumption at 9.90 Wh, making it the most suitable configuration for energy-constrained deployments. Nevertheless, a comprehensive autonomy assessment requires evaluating the power demand against both battery capacity and solar generation.

Within this context, the system is powered by a Li-ion battery with a nominal capacity of 4400 mAh, equivalent to 48.84 Wh at the nominal voltage of 11.1 V. To estimate the average daily solar generation, one year of irradiance data was obtained from the Brazilian National Meteorological Institute (INMET) database. The data was processed alongside the solar panel parameters to estimate the daily energy harvested in watt-hours under two irradiance scenarios. The first scenario, Normal Sunlight, represents the average daily generation computed from the mean annual solar irradiance. The second scenario, Poor Sunlight, represents an extremely conservative low-irradiance condition. This corresponding irradiance threshold was defined using the 1st percentile of the annual daily irradiance distribution, ensuring that 99% of days present equal or higher solar incidence. Since OP1 operates connected to the power grid, solar irradiance has no impact on its autonomy. Therefore, the analysis focuses on OP2 and its Alert variant.

Table 7 summarizes the energy balance and estimated system autonomy across operating modes and irradiance conditions. OP1 operates connected to the local power grid and is therefore capable of sustained long-term operation regardless of solar incidence. In OP2 mode, the energy balance remains positive under normal sunlight, ensuring energetic self-sufficiency. Under poor sunlight, the system maintains operability with a surplus of +0.26 Wh/day in standard measurement mode. In Alert mode, a deficit of −4.13Wh/day is observed, reducing autonomous operation to approximately 12 days based solely on battery capacity. Nevertheless, such an energy deficit would only occur under extreme conditions, such as twelve consecutive days of low solar irradiance. Under complete absence of sunlight, the system remains operational for up to 4.9 days in standard measurement mode and approximately 3.4 days in Alert mode, relying exclusively on battery power.

These results demonstrate that the proposed system is capable of sustained autonomous operation under battery-powered conditions, even in scenarios of reduced solar availability. Therefore, the results confirm the robustness of the proposed solution for long-term deployment in remote environments, where energy constraints and variability in solar resources are critical factors.

### 4.4. Sensor Performance

**Figure 15:**
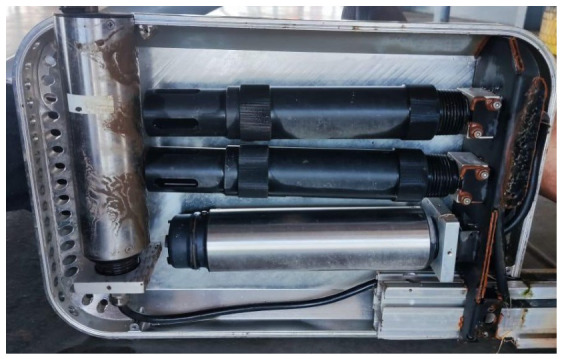
Assessment of sediment accumulation on the sensors.

Figure 15 illustrates the condition of the sensing structure after approximately one month of continuous submersion in the Amazon River. A considerable accumulation of sediments was observed on the external surfaces of both the sensors and the mechanical enclosure, reflecting the high sediment load characteristic of Amazonian river systems. Despite the sediment deposition, no significant degradation or measurement instability was detected during this period, demonstrating the operational robustness of the proposed sensing system under prolonged exposure to sediment-rich environmental conditions.

During the experimental campaign, periodic maintenance and cleaning procedures were performed at monthly intervals to minimize the effects of sediment accumulation and biofouling on the sensing interfaces. According to manufacturer specifications, the sensors typically require recalibration at intervals ranging from one to six months, depending on the sensing principle, as summarized in Table 8.

However, no recalibration procedures were performed during the three-month validation period. Despite this, the monitoring system maintained stable operation and consistent measurements throughout the deployment. These results indicate that, under the tested environmental conditions, monthly maintenance combined with the inherent stability of the sensors was sufficient to ensure reliable performance without the need for recalibration within the observed period.

### 4.5. Web-Based Monitoring Platform

**Figure 16:**
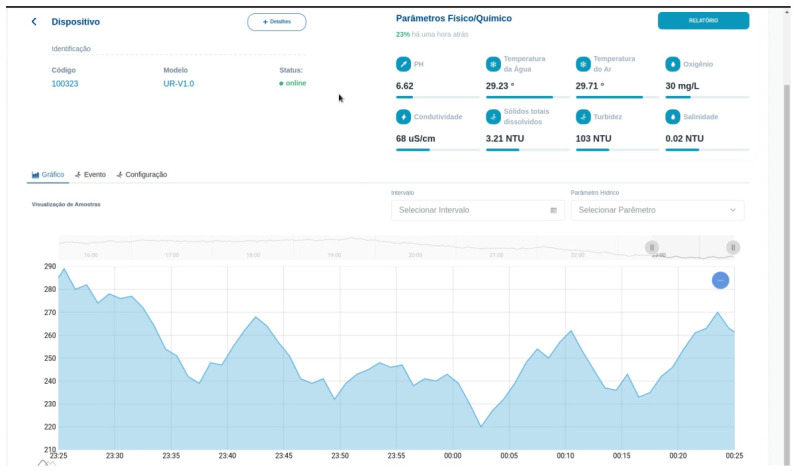
Online control panel displaying real-time water quality parameters.

The web-based monitoring platform enabled the real-time visualization of the data transmitted by the device, providing continuous access to water quality parameters as they were collected in the field. The interface provides both real-time measurements and historical trends through an interactive dashboard, enabling users to monitor the parameters collected by the device. As illustrated in Figure 16, the platform includes graphical tools that support temporal analysis, enabling the identification of variations, trends, and transient events in the monitored dataset.

**Figure 17:**
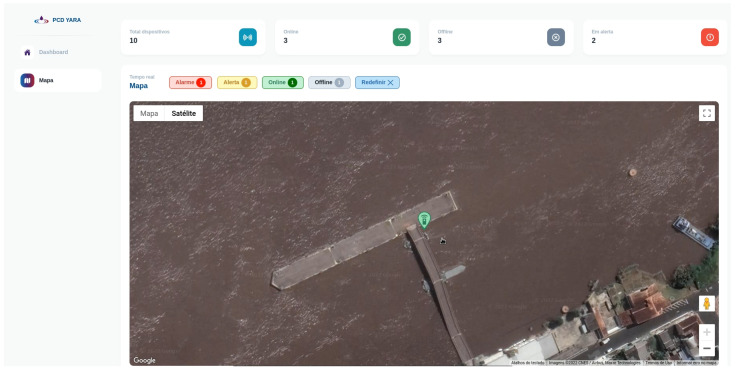
Geospatial visualization of the monitoring platform, showing the exact deployment location of the device at the Port of Parintins, Amazon River.

In addition to real-time monitoring, the platform enables the generation of automatic alerts when anomalous events are detected based on configurable threshold conditions. These threshold values and sampling configurations can be dynamically adjusted by the user, allowing the system to adapt to different hydrological environments and monitoring requirements. Furthermore, the platform integrates geospatial visualization features, enabling users to identify the exact deployment location of the monitoring device, as illustrated in Figure 17, and correlate environmental data with site-specific conditions.

### 4.6. Comparative Analysis of the Reference and Automated Datasets

Based on the datasets described in the previous section, a comparative analysis was conducted to evaluate the consistency, variability, and performance of the automated monitoring system in relation to the reference measurements. The results focus on identifying similarities and differences between the datasets, as well as assessing the reliability of the proposed system under real operating conditions.

#### 4.6.1. pH Data Results

**Figure 18:**
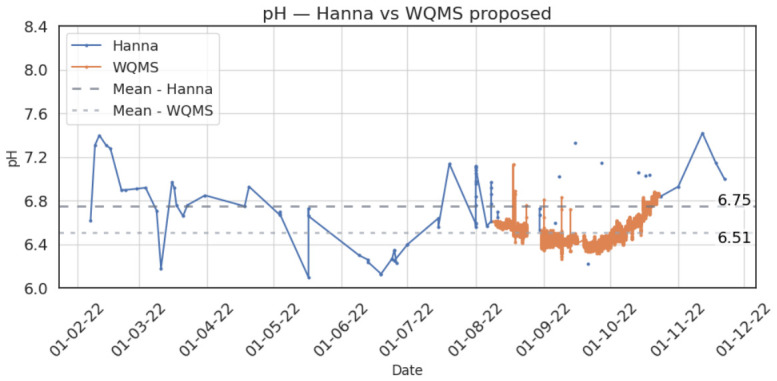
Manually collected pH data vs. automatic device pH data.

The comparative analysis of the pH values obtained from manual measurements and the automated monitoring system, shown in Figure 18, demonstrates strong agreement between both methods, indicating the reliability of the proposed system.

The measured values ranged between 6.4 and 7.1, which is consistent with the slightly acidic to neutral conditions typically observed in the Amazon River, influenced by organic matter decomposition and forest dynamics.

The automated system exhibited lower dispersion and improved temporal stability, highlighting its ability to continuously capture small natural variations without the need for human interference. In contrast, manual measurements showed occasional fluctuations associated with sampling conditions and environmental variability.

#### 4.6.2. Water Temperature Data Results

**Figure 19:**
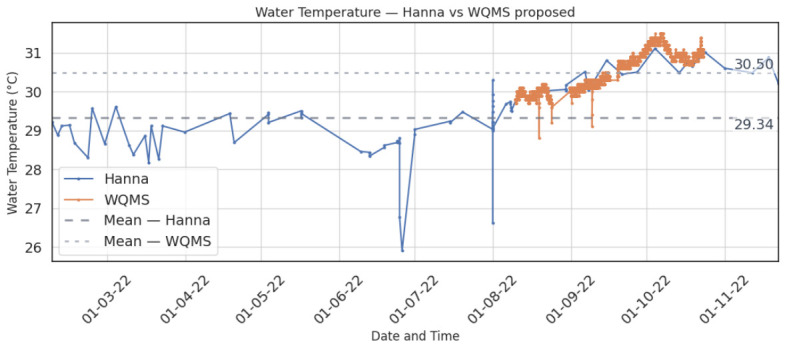
Manually vs. Automatic Water Temperature Data Collection.

The water temperature measurements obtained from manual sampling and the automated system are presented in Figure 19. The results show consistent agreement between both methods, with values typically ranging between 28 °C and 31 °C.

Manual measurements exhibited small fluctuations associated with sampling conditions, including the time of day, sensor handling, and environmental variability. In contrast, the automated system provided a more continuous and stable temperature profile, capturing gradual variations related to solar radiation and the hydrological dynamics.

The automated measurements demonstrated lower dispersion and greater temporal coherence, indicating improved reliability in representing actual environmental conditions. This continuous monitoring capability allows for a more accurate characterization of thermal behavior in the river system.

#### 4.6.3. Turbidity Data Results

The turbidity values obtained by the automated monitoring system showed a wide dynamic range, varying from values close to zero to peaks exceeding 275 NTU. Most measurements remained within the 35 to 60 NTU range, indicating relatively stable sediment conditions throughout most of the monitoring period.

The system successfully captured both gradual variations and abrupt increases in turbidity, which are associated with sediment transport dynamics and hydrological fluctuations typical of the Amazon River. These peaks reflect natural processes such as increased flow velocity, sediment resuspension, and localized disturbances.

It is important to note that occasional zero values were observed in the dataset, which are not physically consistent with natural turbidity conditions. These occurrences are associated with the optical sensing mechanism of the ZDYG-2088-01 digital turbidity sensor, which incorporates an automatic cleaning system at the measurement interface. This mechanism is activated intermittently to prevent fouling and maintain measurement reliability in sediment-rich environments, and during certain acquisition cycles its activation coincided with the data acquisition command, resulting in transient zero readings.

To address this issue, a contextual temporal filtering approach was adopted for the turbidity measurements, as described in Algorithm 1. The time series was scanned to identify contiguous zero-valued segments, referred to as zero-valued events. For each event, the medians of a window of w=3 valid non-zero samples immediately before and after the zero-valued segment were computed. The use of median-based filtering was adopted to reduce the potential bias associated with the indiscriminate removal of zero values, providing a more robust assessment of local temporal behavior and minimizing the influence of outliers or transient fluctuations in the surrounding measurements.

A zero-valued event was preserved only when both local medians were below the near-zero threshold $\tau_{0}=20$ NTU, indicating consistency with a potentially valid low-turbidity condition. Otherwise, the event was classified as an acquisition or sensor artifact and removed.
**Algorithm 1** Temporal consistency filter for zero-valued turbidity artifacts.**Require:** Turbidity time series $X={\left\{\left(t_{i},y_{i}\right)\right\}}_{i=1}^{N}$**Require:** Window size w=3**Require:** Near-zero threshold $\tau_{0}=20$ NTU **Ensure:** Filtered turbidity time series $X_{f}$
 1:Initialize $X_{f}\leftarrow X$ 2:Identify all contiguous zero-valued events$$B_{k}=\left\{i:y_{i}=0\right\},\;k=1,\ldots ,K$$ 3:**for** each zero-valued event $B_{k}$ **do** 4:  Let $i_{s}$ and $i_{e}$ be the first and last indices of $B_{k}$ 5:  Select the *w* valid non-zero samples immediately before $B_{k}$:$$P_{k}=\left\{y_{i}:i<i_{s},\;y_{i}\neq 0\right\}$$ 6:  Select the *w* valid non-zero samples immediately after $B_{k}$:$$Q_{k}=\left\{y_{i}:i>i_{e},\;y_{i}\neq 0\right\}$$ 7:  Compute the local medians:$$m_{k}^{-}=\mathrm{median}\left(P_{k}\right),\;m_{k}^{+}=\mathrm{median}\left(Q_{k}\right)$$ 8:  **if** $m_{k}^{-}\leq \tau_{0}$ **and** $m_{k}^{+}\leq \tau_{0}$ **then** 9:    Preserve the zero-valued event $B_{k}$   ▹temporal behavior is consistent with near-zero turbidity10:  **else**11:    Remove all samples in $B_{k}$ from $X_{f}$   ▹zero-valued event classified as sensor artifact12:  **end if**13:**end for**14:**return** $X_{f}$

After quality control, the final valid turbidity dataset contained 9018 measurements from the original 9914 samples. A total of 896 zero-valued measurements were classified as artifacts and removed, representing 9.06% of the original dataset. Therefore, 90.94% of the turbidity measurements were retained for subsequent analysis.

A potential bias introduced by this filtering procedure is the underrepresentation of very low turbidity values, since all zero-valued events were removed. This may slightly increase statistical metrics such as the minimum, mean, and lower percentiles. However, this effect was mitigated by applying a temporal consistency criterion instead of removing zero values unconditionally. Zero-valued events would have been preserved if the medians of the surrounding valid measurements were below the near-zero threshold $\tau_{0}=20$ NTU. Since no event satisfied this condition, the removed samples were considered more consistent with acquisition or sensor artifacts than with physically plausible low-turbidity conditions.

**Figure 20:**
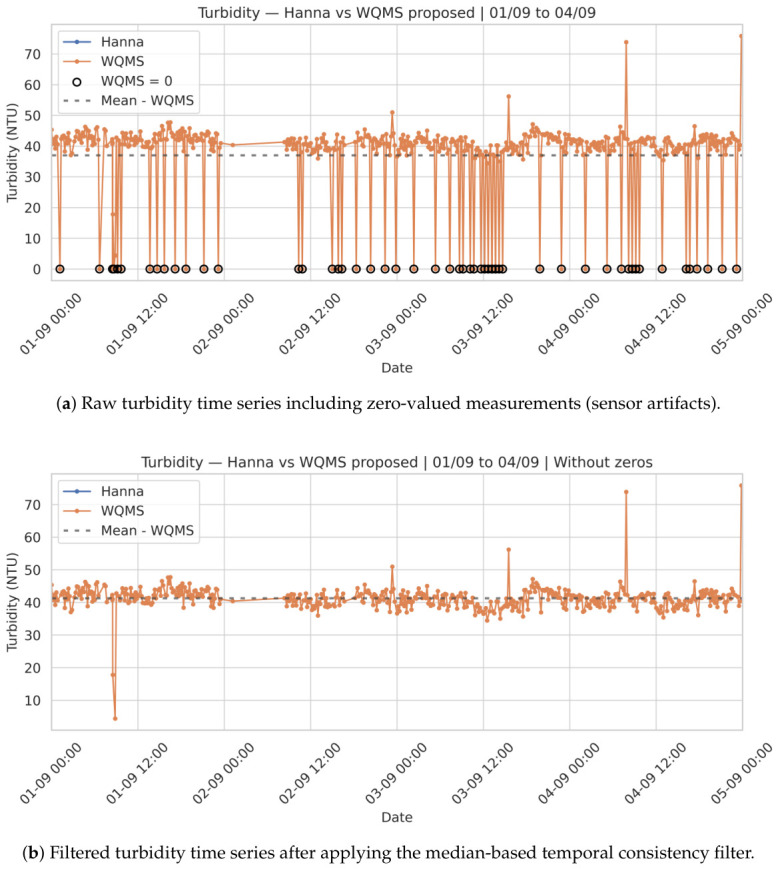
Comparison between raw and filtered turbidity measurements over a representative time window. In (**a**), frequent zero-valued readings associated with the sensor cleaning mechanism produce abrupt and physically inconsistent drops. In (**b**), the proposed median-based temporal filter removes these artifacts while preserving the underlying temporal dynamics, including trends, mean levels, and natural variability.

Figure 20 presents a comparative time-series visualization of turbidity measurements over a representative multi-day period, showing both the raw dataset (including zero-valued readings) and the filtered dataset obtained after applying the proposed median-based temporal consistency filter.

The comparison demonstrates that the proposed filtering approach effectively eliminates spurious zero-valued artifacts without altering the natural variability of the turbidity signal. The filtered time series maintains the overall trend, mean level, and short-term fluctuations observed in the raw data, while removing only the physically inconsistent measurements.

Importantly, the filter does not suppress legitimate turbidity variations, such as gradual changes or peak events associated with sediment transport dynamics. Instead, it selectively removes isolated or contiguous zero-valued segments that are inconsistent with the surrounding measurements, based on a temporal consistency criterion using local medians.

**Figure 21:**
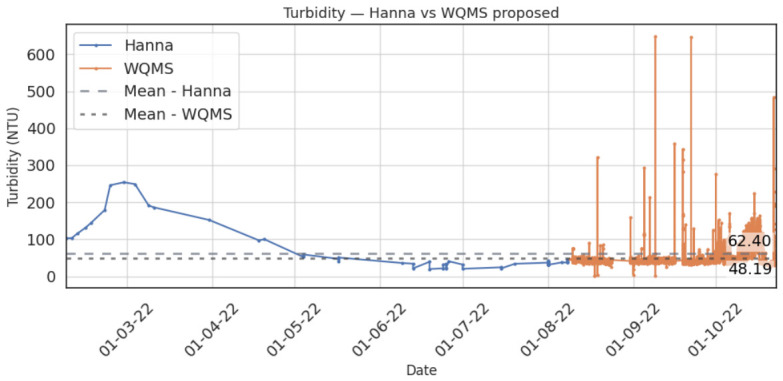
Manually collected turbidity data vs. automatic device turbidity data.

Compared to manual measurements, the automated system provides a continuous representation of turbidity variations, enabling the identification of temporal patterns that cannot be captured through discrete sampling approaches. The effect of the filtering procedure and the comparison between manual and automated measurements can be observed in Figure 21.

**Figure 22:**
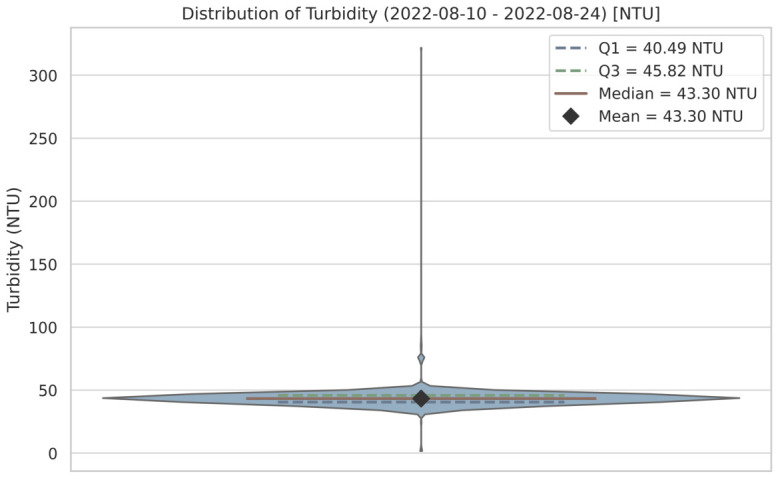
Statistical distribution of turbidity measurements acquired between 10 August 2022 and 24 August 2022 during the monitoring campaign, highlighting the median, mean, and interquartile range (Q1–Q3).

To further complement the statistical analysis of the turbidity measurements acquired by the automated monitoring system, a distribution-level evaluation was performed using the complete dataset collected between 10 August 2022 and 24 August 2022. Figure 22 presents the statistical distribution of the turbidity values, highlighting the mean, median, and interquartile range (Q1–Q3). This analysis complements the point-wise error metrics by providing additional insight into the variability and central tendency of the monitored parameter under real environmental conditions in the Amazon River.

#### 4.6.4. Electrical Conductivity (EC) Data Results

**Figure 23:**
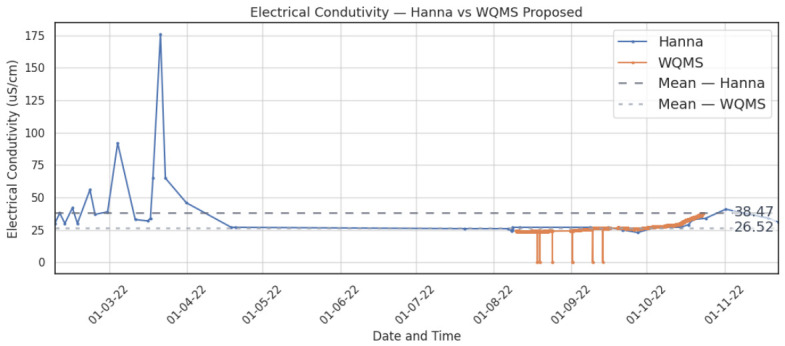
Manually collected EC data vs. automatic device EC data.

A comparative analysis of electrical conductivity (EC) measurements obtained using manual and automated methods is presented in Figure 23. The results show strong agreement between the two datasets, with values remaining within the expected range for the Amazon River.

The conductivity values were predominantly between 40 and 120 µS/cm, which is characteristic of freshwater environments with low mineralization. This behavior reflects the high dilution capacity of the river and the limited presence of dissolved salts.

Manual measurements exhibited occasional peaks, likely influenced by localized conditions such as sediment concentration and variations near the riverbank. In contrast, the automated system showed greater temporal consistency, capturing gradual variations with reduced measurement noise.

#### 4.6.5. Air Temperature Data Results

**Figure 24:**
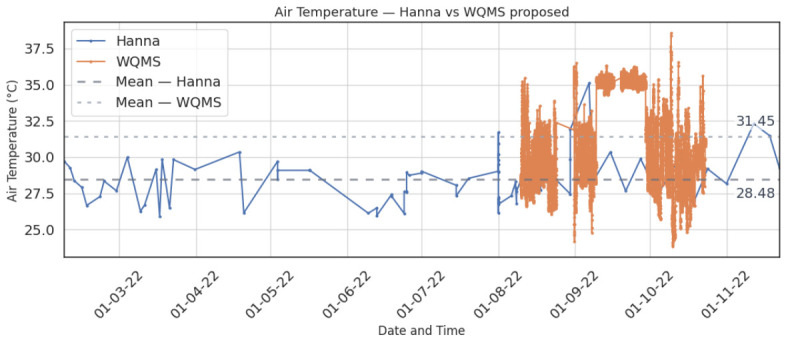
Manual vs. Automatic Air Temperature Data Collection.

While the manual measurements were conducted once per day, providing a limited temporal representation of environmental conditions, the automated system performs data acquisition at short and regular intervals, enabling the analysis of a broader temporal window throughout the day. This approach reveals significant intraday variability that is not captured by the manual method. Figure 24 compares the air temperature measurements obtained using the manual reference instrument and the proposed automated monitoring system.

The data show pronounced temperature oscillations over daily cycles, with values ranging approximately between 25 °C and 38 °C, reflecting the strong influence of solar radiation, atmospheric humidity, and local climatic conditions typical of the Amazon region. Due to its low sampling frequency, the manual dataset tends to smooth out or completely miss these short-term variations.

In contrast, the automated system captures both the overall temperature trend and rapid intraday fluctuations, providing a more detailed and representative characterization of environmental conditions.

#### 4.6.6. Statistical Comparison

To evaluate the performance of the proposed monitoring system, a statistical comparison was conducted against reference measurements obtained using a manual probe. The analysis considered point-wise error metrics, including mean absolute error (MAE), root mean square error (RMSE), and bias, computed from temporally matched observations within the common measurement interval.

Due to the data acquisition process, the number of paired samples is limited. The reference measurements were collected manually, typically once per day, while the proposed system operated continuously during its deployment period. For temporal matching, manual and automated measurements were considered aligned when they were acquired on the same calendar day. For each manual Hanna measurement, the corresponding WQMS measurement available on the same day was used for comparison. After applying this same-day matching criterion, the maximum observed temporal difference between the retained manual and automated measurements was approximately 3 min. Therefore, the 3-min value represents the maximum observed separation among the matched pairs, rather than an additional exclusion threshold. Measurements without a corresponding same-day record in the common measurement interval were not included in the point-wise error analysis.

Using this matching procedure, N=12 valid manual–automated pairs were obtained. Thus, *N* refers to the number of valid same-day matched pairs available after overlap verification and data quality control, and not to the total number of days covered by the manual campaign or by the continuous WQMS deployment.

A distribution-level comparison was performed using the temporally matched samples (N=12). Table 9 presents the statistical comparison between manual and automated measurements, including mean, median, interquartile range (IQR), and total range.

Table 9 shows that water temperature exhibits the highest agreement between the datasets, with nearly identical mean, median, variability, and range values. For pH, the central tendency remains consistent, although the WQMS data present reduced variability. In contrast, turbidity and air temperature show higher variability in the WQMS dataset, indicating greater sensitivity to environmental fluctuations and dynamic conditions.

In addition to the distribution-level analysis, point-wise error metrics were computed using the same temporally matched samples (N=12). Table 10 summarizes the mean absolute error (MAE), root mean square error (RMSE), and bias for each parameter.

The results indicate that water temperature presents the highest agreement between the systems, with negligible bias and nearly identical distribution characteristics. For pH, a moderate negative bias suggests a tendency toward underestimation, although the overall distribution remains consistent. Turbidity exhibits higher error and variability, with the automated system capturing a broader range of values, reflecting its higher temporal resolution and sensitivity to environmental fluctuations. Air temperature shows the largest deviation, with systematic overestimation and increased variability, likely influenced by sensor exposure and installation conditions. It is important to note that, for highly dynamic environmental parameters such as turbidity and air temperature, the reported error metrics combine both instrument-related differences and natural environmental variability and therefore should not be interpreted solely as indicators of measurement inaccuracy. Overall, the results demonstrate that the proposed system provides reliable measurements for stable parameters while effectively capturing the natural variability of more dynamic environmental conditions.

### 4.7. Correlation Analysis

**Figure 25:**
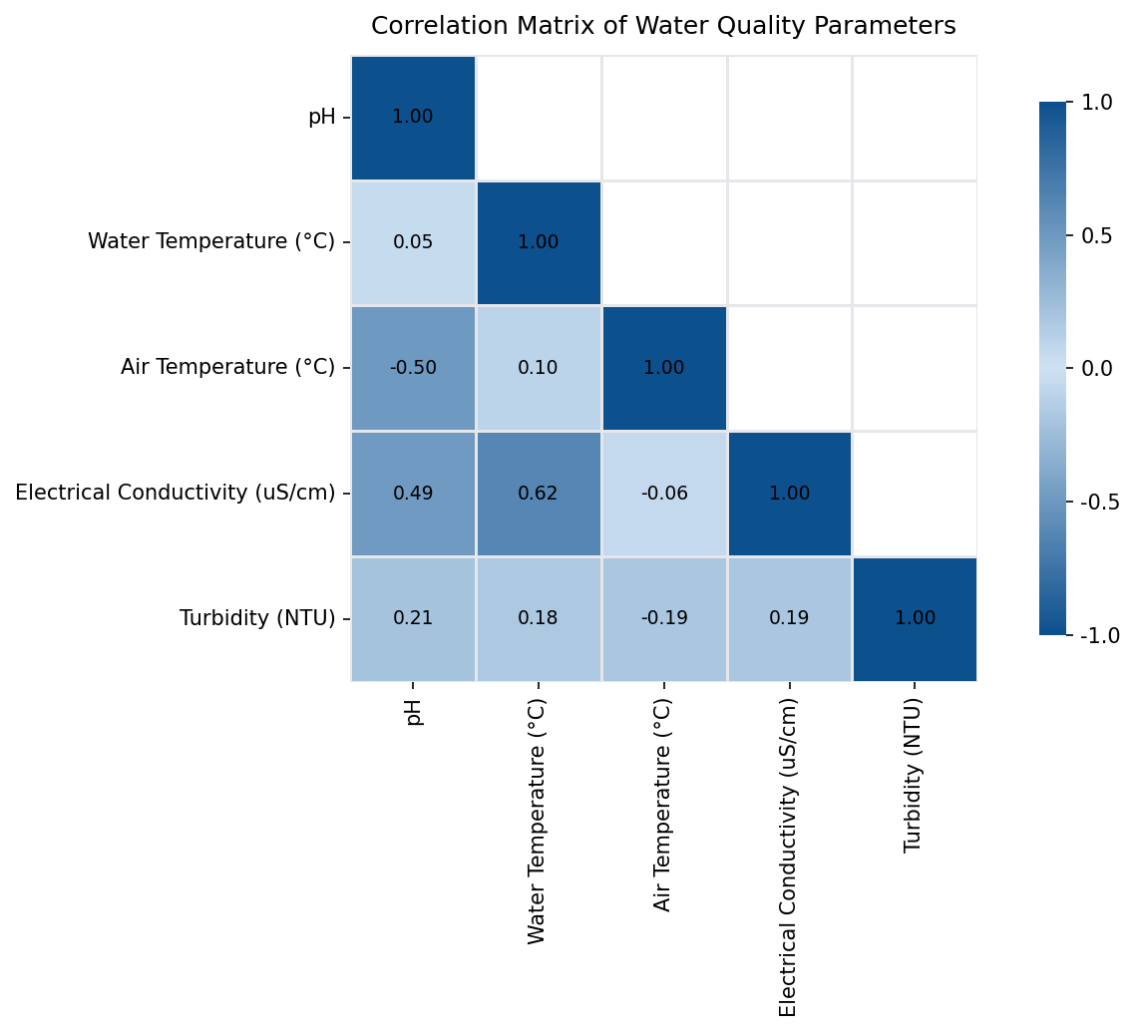
Pearson correlation matrix of the monitored water quality parameters, including pH, water temperature, air temperature, electrical conductivity, and turbidity.

Figure 25 presents the correlation matrix of the monitored water quality parameters, providing insights into the relationships among the measured variables.

A moderate positive correlation was observed between electrical conductivity and water temperature (r = 0.62), suggesting that temperature variations may influence ionic mobility and the concentration of dissolved minerals. Additionally, electrical conductivity exhibited a moderate positive correlation with pH (r = 0.49), which may be associated with variations in dissolved ion concentration affecting the acid-base balance of the water.

A moderate negative correlation was identified between pH and air temperature (r = −0.50), indicating that atmospheric conditions may indirectly influence water chemistry through temperature-related environmental interactions. However, this relationship should be interpreted with caution, as it may reflect transient environmental conditions rather than a direct causal relationship.

Turbidity exhibited weak correlations with all other monitored parameters, with correlation coefficients ranging from −0.19 to 0.21. This behavior suggests that suspended solids in the water are primarily influenced by hydrodynamic processes, such as sediment transport and flow variations, rather than by direct interactions with the measured physicochemical variables.

Overall, the correlation analysis indicated limited linear dependency among the monitored variables, reinforcing the importance of continuous monitoring for capturing the complex and nonlinear interactions that characterize water quality dynamics in large tropical river systems such as the Amazon River.

## 5. Discussion

The results presented in Table 4 provide a comprehensive comparison between the automated monitoring system (WQMS) and the reference commercial instruments (Hanna), highlighting both the reliability of the proposed system and the environmental behavior of the monitored parameters. For pH, water temperature, electrical conductivity, and air temperature, no anomalous values were identified in either dataset, with the WQMS recording 9914 normal measurements and the manual dataset confirming this trend with 146, 140, and 140 measurements, respectively. This consistency indicates that these parameters exhibited relatively stable behavior in the monitored region and that the proposed system was capable of accurately reproducing the expected environmental conditions.

In contrast, turbidity exhibited a significantly different pattern, with 370 anomalous measurements out of 9914 (approximately 3.7%) in the automated dataset, compared to 17 anomalous values out of 132 measurements (approximately 12.9%) in the manual dataset. This difference is primarily associated with the higher temporal resolution of the automated monitoring system, which enabled a more detailed characterization of transient hydrological events. The automated system recorded turbidity values ranging from near-zero to peaks exceeding 275 NTU, with most measurements concentrated between 35 and 60 NTU, indicating relatively stable sediment conditions throughout most of the monitoring period. The system successfully captured both gradual variations and abrupt increases in turbidity associated with sediment transport dynamics, flow velocity variations, sediment resuspension, and localized disturbances typical of the Amazon River.

It is important to note that 896 measurements (approximately 9.04% of the complete turbidity dataset) presented zero values, which are not physically consistent with the environmental conditions observed in the monitored river section. These occurrences were associated with the automatic cleaning mechanism integrated into the ZDYG-2088-01 optical turbidity sensor. During specific acquisition cycles, the activation of the cleaning mechanism coincided with the measurement routine, generating transient zero readings. Although these events were identified as transient artifacts associated with sensor maintenance cycles, they were considered during the interpretation of the turbidity dataset and highlight practical challenges related to long-term automated monitoring in sediment-rich river environments. To mitigate potential bias from indiscriminate data removal, a contextual temporal filtering approach was applied to selectively identify and remove only non-representative zero-valued events based on the surrounding measurement behavior.

Compared with manual measurements, the automated system provided a continuous representation of turbidity dynamics, enabling the identification of temporal patterns and short-term fluctuations that cannot be captured through conventional discrete sampling approaches. The lower dispersion and improved temporal consistency of the automated measurements further demonstrate the operational robustness of the proposed monitoring system under sediment-rich tropical river conditions.

From a system perspective, the complete monitoring solution demonstrated robust and reliable operation under real environmental conditions. The mechanical enclosure effectively protected the sensors against external stressors, including high sediment loads, water currents, and debris impact, ensuring stable operation throughout the deployment period. The device maintained continuous operation and data acquisition, confirming the effectiveness of the proposed design for long-term monitoring in harsh environments. Additionally, the experimental deployment provided important practical insights into the operational limitations of electrochemical sensing devices in remote environments. During the deployment phase, an issue was identified with the dissolved oxygen (DO) sensor, preventing the acquisition of reliable automated measurements for this parameter. Preliminary analysis suggested that the issue was likely associated with electrical instability during the communication testing stage performed prior to field deployment, including possible grounding (GND) inconsistencies in the communication interface, potentially affecting the electronic circuitry of the sensing module and compromising its operation. Although the failure occurred before long-term field operation, this experience highlighted the importance of validating electrical integration, grounding stability, and communication robustness prior to deployment. These observations provide valuable practical insights for future implementations of automated water quality monitoring platforms operating under challenging tropical river conditions.

The web-based platform further enhanced the system functionality by enabling real-time data visualization, anomaly detection, parameter configuration, and geolocation tracking. These features significantly improved data accessibility and situational awareness, allowing users to remotely monitor environmental conditions and respond more efficiently to environmental variations. The integration of these functionalities demonstrates that the system operates as a complete monitoring solution, extending beyond data acquisition to provide actionable environmental insights.

The autonomy analysis showed that the system can sustain continuous operation under normal and reduced solar irradiance conditions. In OP2 mode, the system maintained a positive energy balance even under poor sunlight, while the Alert configuration remained operational for approximately 12 days relying on battery capacity under the same low-irradiance scenario. These results indicate that the proposed energy-aware configuration is suitable for remote deployments where solar availability may vary significantly.

In addition to its technical performance, the proposed monitoring system represents a relevant contribution to water quality management in the Amazon region. The availability of continuous, high-resolution data enables a more accurate assessment of environmental conditions, supporting the application of regulatory frameworks such as CONAMA Resolution No. 357/2005. In this context, the proposed system supports context-aware environmental monitoring, improving the interpretation of water quality data and enabling more informed and effective decision-making processes for regional environmental management.

## 6. Conclusions

This study presented the development and experimental validation of an IoT-based platform for continuous water quality monitoring deployed under real environmental conditions in the Amazon River. The results demonstrated that the proposed system was capable of reliably monitoring key water quality parameters, including pH, turbidity, electrical conductivity, and temperature, while maintaining continuous operation under challenging riverine conditions characterized by high sediment loads, hydrological variability, and limited communication infrastructure.

During the experimental campaign, the system generated approximately 49,570 measurements, providing high temporal resolution environmental data and enabling the observation of dynamic processes associated with sediment transport and intra-day environmental variability. Compared with conventional manual sampling approaches, the automated monitoring platform provided improved measurement consistency and continuous environmental observation, contributing to a more representative characterization of water quality dynamics in the monitored region.

The successful long-term deployment confirmed the robustness of the integrated monitoring architecture, including the embedded sensing platform, LoRa communication infrastructure, web-based monitoring interface, and mechanical protection structure designed for operation in sediment-rich tropical river environments. In particular, the mechanical enclosure demonstrated satisfactory resistance to prolonged exposure under Amazon River conditions, maintaining sensor operation even under continuous sediment accumulation and environmental stress.

Beyond the individual technological components, the main contribution of this work lies in the holistic integration and extensive field validation of an environmental IoT monitoring platform operating in a highly dynamic and infrastructure-limited tropical environment. This directly addresses the lack of integrated and field-validated monitoring frameworks identified in the literature, particularly for remote and highly dynamic tropical river systems.

The energy analysis confirmed that the system is capable of sustained autonomous operation under varying solar irradiance conditions, directly addressing one of the key challenges associated with long-term environmental monitoring in remote tropical regions. The ability to maintain operation under both typical and adverse energy conditions reinforces the practical applicability of the proposed solution.

From a water resource management perspective, the proposed platform contributes to continuous environmental assessment aligned with regulatory frameworks such as CONAMA Resolution No. 357/2005, supporting anomaly identification, environmental interpretation, and data-driven decision-making processes. The proposed solution also demonstrates strong potential for scalability and replication in other remote and environmentally sensitive regions where conventional monitoring approaches remain limited.

Overall, this study demonstrates that integrated IoT-based monitoring platforms can provide a practical and scalable solution for continuous environmental monitoring, contributing to the advancement of autonomous sensing technologies and supporting sustainable water resource management in complex and underserved regions.

## 7. Future Work

Future work will focus on expanding the functionality, robustness, and applicability of the proposed monitoring system, addressing both technological improvements and environmental challenges associated with long-term operation in remote riverine environments. The following research lines are proposed:

- Predictive environmental analysis: The integration of machine learning techniques for forecasting water quality variations based on historical and real-time measurements, enabling predictive environmental assessment and supporting early identification of anomalous conditions.
- Energy consumption optimization: The implementation of low-power operation strategies and firmware optimizations aimed at reducing energy consumption, extending battery autonomy, and improving long-term operation under low solar irradiance conditions. Future efforts will include a detailed profiling of energy consumption across system components and operational states, enabling the identification of major power contributors and the development of targeted optimization strategies, particularly for sleep-mode efficiency and communication routines.
- Floating deployment platform: The development of a floating mechanical structure to enable flexible installation in river environments without fixed infrastructure, increasing deployment scalability across diverse hydrological conditions.
- Satellite communication integration: The incorporation of satellite-based communication technologies to ensure reliable data transmission in areas without terrestrial connectivity, addressing a key limitation for monitoring in the Amazon region.
- Automated dissolved oxygen measurement: The inclusion of a new dissolved oxygen (DO) sensor to enable automated data acquisition.
- Field validation of threshold-based alert mode: Experimental validation of the Threshold Alert Mode under real environmental conditions, including verification of alert triggering behavior when predefined limits are exceeded, as well as the dynamic increase in acquisition and transmission frequency during significant environmental variations.

## Figures and Tables

**Table 2:** Specifications of the monitoring sensors.

Parameter	Brand	Location	Model	Description	Resolution
pH	BOQU Instruments	Shanghai, China	BH-485-pH Digital pH Sensor	Indicator of the acidity of the medium.	±0.01
Turbidity	BOQU Instruments	Shanghai, China	ZDYG-2088-01 Digital Turbidity Sensor	Indicator of the amount of suspended particles in the water.	±0.01 NTU
Electrical Conductivity	BOQU Instruments	Shanghai, China	BH-485-EC Digital Conductivity Sensor	Indicator of the concentration of dissolved minerals and conductive substances in water; also used for water temperature measurement.	±0.1 μS/cm
Air Temperature	E + E Elektronik	Engerwitzdorf, Austria	EE060	Parameter that affects dissolved oxygen concentration, pH, electrical conductivity, and biological activity.	±0.5 °C
Dissolved Oxygen	BOQU Instruments	Shanghai, China	BH-485-DO Digital DO Sensor	Used to determine the concentration of dissolved oxygen in water.	±3%

**Table 3:** Reference limits for water quality parameters based on CONAMA Resolution No. 357/2005.

Parameter	Limit	Classification Criterion
pH	6.0–9.0	Values outside this range are classified as anomalous
Dissolved Oxygen (DO)	≥5 mg/L	Values below this threshold are classified as anomalous
Turbidity	≤100 NTU	Values above this threshold are classified as anomalous
Electrical Conductivity	Not defined	Evaluated based on deviations from expected behavior
Water Temperature	Not defined	Evaluated based on environmental variation and stability
Air Temperature	Not defined	Used as a reference parameter for environmental conditions

**Table 4:** Comparison of normal and anomalous measurements between WQMS and Hanna instruments.

Parameter	WQMS			Hanna		
	Total	Anomalous	Normal	Total	Anomalous	Normal
pH	9914	0	9914	146	0	146
Water Temperature	9914	0	9914	140	0	140
Turbidity	9914	370	9544	132	17	117
Electrical Conductivity	9914	0	9914	140	0	140
Air Temperature	9914	0	9914	140	0	140

**Table 5:** Device current consumption per system states.

State	Mean Current	Time Duration	Current Spike
Measurement	185 mA	60 s	420 mA
Transmission	100 mA	1 s	120 mA
Sleep	20 mA	—	—

Current was measured at 11.1 V nominal battery voltage. Sleep refers to any period without active measurement or transmission.

**Table 6:** Energy consumption per operational modes.

Mode	Meas./h	KA/h	Wh/h	Wh/Day
OP1	30	30	1.15	27.48
OP1 (Alert)	60	30	2.06	49.51
**OP2**	6	30	0.41	9.90
OP2 (Alert)	12	30	0.60	14.30

Meas./h: Measurements per hour; KA/h: Keep Alive transmissions per hour.

**Table 7:** Battery autonomy under different solar irradiance conditions.

Mode	Wh/Day	Normal Sunlight (43.65 Wh/Day)	Poor Sunlight (10.16 Wh/Day)	No Sunlight
OP1 & OP1 (Alert)	27.48 49.51	On grid	On grid	On grid
OP2	9.90	Continuous +33.75 Wh/day	Continuous +0.26 Wh/day	4.9 days −9.9 Wh/day
OP2 (Alert)	14.30	Continuous +29.35 Wh/day	11.82 days −4.13 Wh/day	3.4 days −14.3 Wh/day

Battery capacity: 4400 mAh (48.84 Wh at 11.1 V). Continuous: Daily energy balance is positive, indicating that the system remains energetically self-sufficient. Poor Sunlight: Conservative irradiance condition representative of very low solar incidence days in Manaus.

**Table 8:** Recommended calibration intervals for the monitoring sensors.

Sensor	Recommended Calibration Interval
BH-485-pH	1–3 months
ZDYG-2088-01 Turbidity	3–6 months
BH-485-EC	3–6 months
EE060	12 months or no frequent periodic calibration required
BH-485-DO	1–3 months

**Table 9:** Distribution-level comparison between manual reference measurements and WQMS data.

Parameter	Mean Hanna.	Mean WQMS	Median Hanna.	Median WQMS	IQR Hanna.	IQR WQMS	Range Hanna.	Range WQMS
pH	6.85	6.52	6.89	6.52	0.40	0.16	1.11	0.33
Turbidity (NTU)	41.14	35.24	40.90	40.22	2.98	9.72	17.00	54.68
Water Temperature (°C)	30.43	30.44	30.50	30.55	0.65	0.68	1.31	1.50
Air Temperature (°C)	29.10	31.28	28.62	30.18	2.36	3.51	8.15	8.61

**Table 10:** Comparison of error metrics between the proposed system (WQMS) and the reference probe.

Parameter	MAE	RMSE	Bias	N
Air Temperature (°C)	2.95	3.57	2.18	12
Water Temperature (°C)	0.18	0.20	0.01	12
Turbidity (NTU)	12.99	20.09	−5.90	12
pH	0.36	0.44	−0.33	12

## Data Availability

The data presented in this study are available on request from the corresponding author.
